# Design and analysis issues in gene and environment studies

**DOI:** 10.1186/1476-069X-11-93

**Published:** 2012-12-19

**Authors:** Chen-yu Liu, Arnab Maity, Xihong Lin, Robert O Wright, David C Christiani

**Affiliations:** 1 Environmental and Occupational Medicine and Epidemiology Program, Department of Environmental Health, Harvard School of Public Health, Boston, MA, USA; 2 Institute of Environmental Health, College of Public Health, National Taiwan University, Taipei, Taiwan; 3 Department of Biostatistics, Harvard School of Public Health, Boston, MA, USA; 4 Department of Statistics, North Carolina State University, Raleigh, NC, 27695, USA; 5 Department of Preventive Medicine, Mount Sinai School of Medicine, New York, NY, USA; 6 Department of Medicine, Massachusetts General Hospital/Harvard Medical School, Boston, MA, USA

**Keywords:** Gene-environment, Interactions, Expanded environmental genomic disease paradigm, Critical developmental windows, Genome-wide, Epigenetics

## Abstract

Both nurture (environmental) and nature (genetic factors) play an important role in human disease etiology. Traditionally, these effects have been thought of as independent. This perspective is ill informed for non-mendelian complex disorders which result as an interaction between genetics and environment. To understand health and disease we must study how nature and nurture interact. Recent advances in human genomics and high-throughput biotechnology make it possible to study large numbers of genetic markers and gene products simultaneously to explore their interactions with environment. The purpose of this review is to discuss design and analytic issues for gene-environment interaction studies in the “-omics” era, with a focus on environmental and genetic epidemiological studies. We present an expanded environmental genomic disease paradigm. We discuss several study design issues for gene-environmental interaction studies, including confounding and selection bias, measurement of exposures and genotypes. We discuss statistical issues in studying gene-environment interactions in different study designs, such as choices of statistical models, assumptions regarding biological factors, and power and sample size considerations, especially in genome-wide gene-environment studies. Future research directions are also discussed.

## Introduction

Although some diseases are predominantly environmental or genetic, both environmental and genetic factors play an important role in most common or complex human diseases. One of the major challenges of exploring mechanisms and treatment of complex diseases is that neither purely environmental factors, nor purely genetic factors can fully explain the observed estimates of disease incidence and progression. To correctly model risk estimates, we must measure genetics and environment together in the same studies. Recent advances in human genomics have made it possible to study tens of thousands of genes simultaneously and incorporate their interactions with the environment. In this review, we discuss design and analysis issues for gene-environmental interactions studies.

Traditional study designs have been used to study gene-environment interaction, including cohort and case–control studies. However some designs tend to favor the measurement of genetic over environmental factors. For example, because genotypes do not vary over time, case–control studies have been more common than cohort studies for studying genetic associations. Genotypes can always be presumed to precede phenotype and the efficiency of a case–control design over a cohort design in determining genetic main effects is well known. Several other methods, such as family-based and case-only studies have also been used, but like case–control studies, sampling is still predicated on the presence of the disease phenotype. Some of the earlier discussions of these study designs in studying genes and environment can be found in Caparaso et al. [1], Langholz et al. [2] and Garcia-Closas et al. [3]. We focus below on design and analysis issues in studying gene-environment interactions in environmental epidemiological studies including recent developments.

## How genetic and environmental factors work together to affect phenotypes

**Figure 1:**
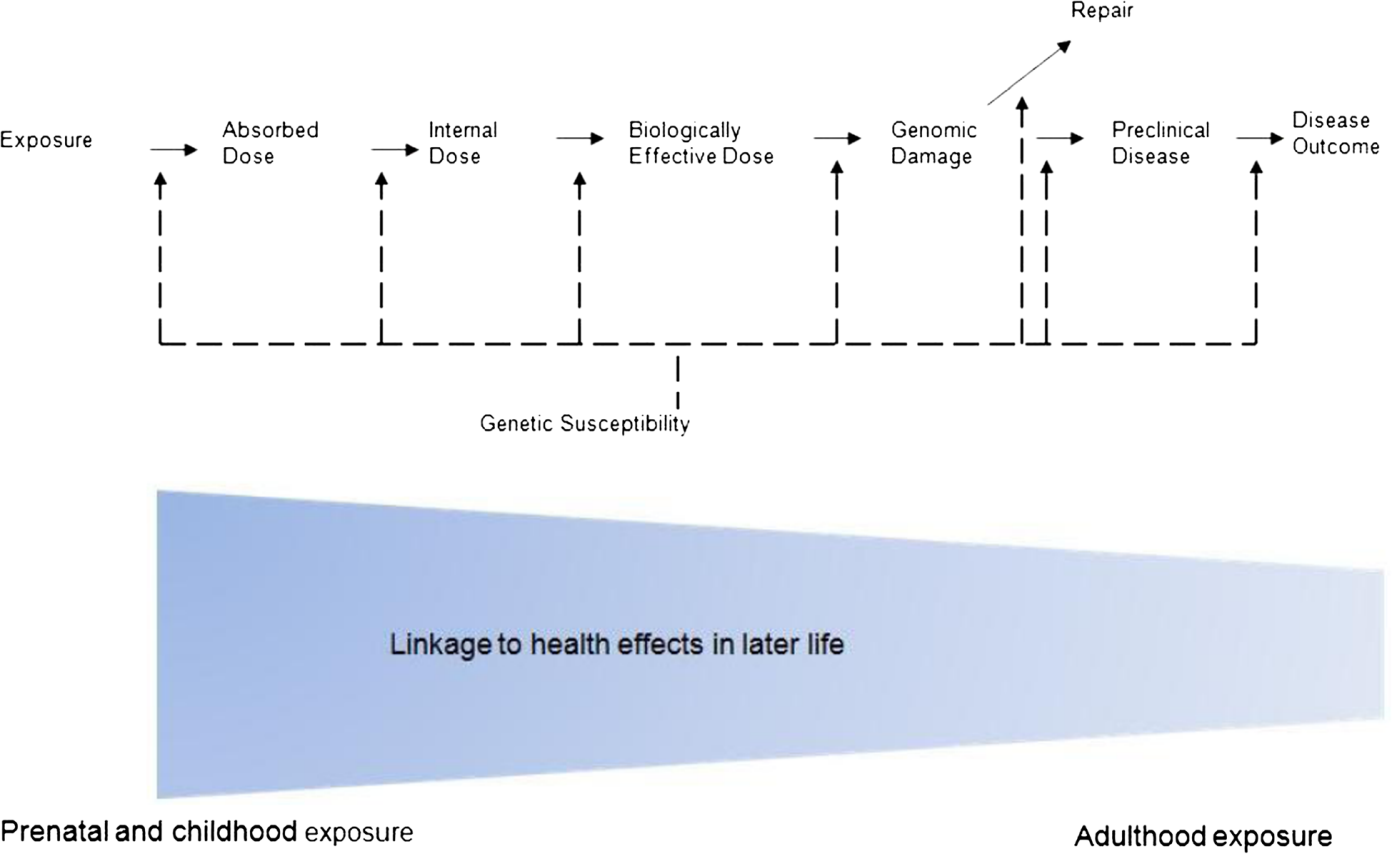
**The integrated paradigm of genetic susceptibility in environmental disease development in different life stage.** The exposure effects during critical developmental period (prenatal and childhood exposure) are highlighted

The detection of a gene-environment interaction likely depends on more than the measurement of a genotype and an exposure. Even a cumulative index of exposure to the environmental factor may not be sufficient. It is well known that environmental exposures vary over time, but what is frequently not considered is that gene expression also varies over time. Human development consists in large part on the timed expression and silencing of specific genes in specific cells at specific life stages. From a purely biological perspective it is difficult to conceive of a gene-environment interaction occurring when the environmental exposure occurs during a life stage when the gene is not expressed. An overly simplistic example might be a chemical which inhibits growth by interacting with a variant in a growth factor gene. Chemical exposure at age 25 years cannot affect final height, while exposure in childhood can. In the field of toxicology, the concept of critical developmental windows of exposure has developed over the last 30 years. Rather than considering a chemical as having a single dose response curve for toxicity, chemicals appear to have different dose response curves depending on the life stage at which exposure occurs. For example, i*n utero* diethylstilbesterol exposure is associated with vaginal cancer in offspring, while mothers who took the drug do not appear to be at risk. In effect, gene-environment interaction may be conceived as a 3-way interaction, in which the time of the exposure is the 3^rd^ factor. Alternatively one can consider environmental exposure as a time-varying covariate and study gene and time-varying-environment interactions by considering lag effects. As shown in Figure 1, we have integrated the time of the exposure in the paradigm by highlighting different exposure effects during each life stage. Direct measures of personal exposure, in particular biomarkers of exposure, provide insights into chemical, social or physical factors to specific individuals. The use of biomarkers of effect in epidemiologic studies allows researchers to study intermediate phenotypes (Figure 1) [4-6]. For example, glycosylated hemoglobin, a measure of chronic serum glucose, can be used to study diabetic risk factors with more power than a study focused on clinical diabetes. In spite of these potential advantages, the results of biomarker measurements sometimes can confuse the investigators a lot. Different conclusions may arise due to the differences of specimen kinds, collection and processing methods, laboratory error, and individual variation in the biomarker levels over time [7]. The usefulness of a biomarker is strongly depending on the specificity, sensitivity, assay reliability, and cost [8].

Another approach, instead of studying unknown effects, is by taking advantage of the established associations between genetic variations and exposure intermediate phenotypes. These genetic variations can mimic the modifiable exposure effects and serve as a surrogate to test the association between exposure and disease. This method has been referred to as ‘Mendelian randomization’, which provides an approach for making causal inferences about the exposure by using the nature of randomly assigned genotypes from parents to offspring before conception [9,10]. However, as well with all genetic association studies, potential confounding effects by population stratifications and other limitations can still occur [10,11]. Careful study conduction and thorough verification remains essential before considering the causality.

### Epigenetics

**Figure 2:**
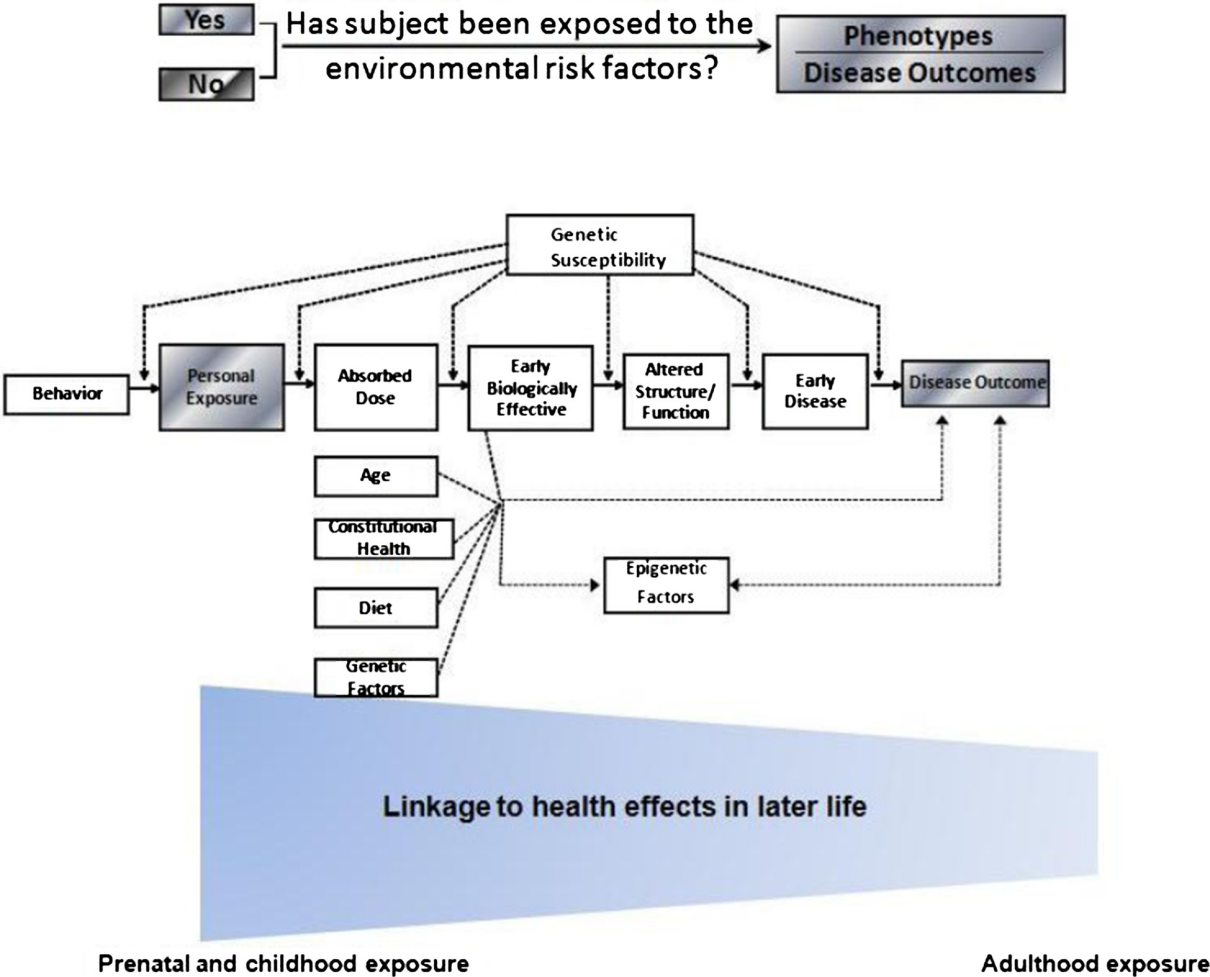
The expanded environmental genomic paradigm

The role of epigenetics has been increasingly recognized as a mechanism of gene-environment interaction. Epigenetics refers to changes in gene function without altering DNA sequence. These changes may last for several generations [12]. Epigenetic mechanisms include alterations in DNA methylation, histone modification, and microRNA [13,14]. The toxic effects of exposure for several environmental chemicals, such as metals, particulate air pollution, benzene, endocrine-disrupting chemicals and reproductive toxicants, have been found to be mediated by epigenetic mechanisms [15]. Epigenetic alterations may be induced by environmental exposure, particularly in early development [16]. This field remains particularly compelling because a number of epigenetic events have been recognized as tissue-specific and reversible, which may help explain why exposures affect specific organs and the complexity of individual susceptibility among the exposed population. Epigenetic data, such as DNA methylation, can also be collected for each of the study designs described above. Epigenetic modifications provide a plausible link between the environment and alterations in gene expression that might lead to change of disease phenotypes. An increasing number of animal studies provide evidence of the role of environmental epigenetics both in disease susceptibility and in heritable environmentally induced transgenerational alterations in phenotype [17]. Thus, incorporating and analyzing epigenetic data in G-E statistical analysis has become immensely important. Epigenetic mechanisms in somatic cells also provide a potential explanation of how early life environmental exposures can program long-term effects in chronic disease susceptibility[18,19]. This expanded environmental genomic paradigm is shown in Figure 2.

## Study design issues

### Confounding and selection bias

When designing epidemiologic studies, issues of feasibility, efficiency, expense, and potential sources of bias must be considered. Perhaps the most feasible and efficient design is the case–control design, especially when studying rare diseases. A *case–control* study is conducted to collect data on environmental exposures retrospectively, and collects biomarkers after disease diagnosis of the cases. While genotypes are static and not prone to differential bias, the assessment of environment retrospectively is fraught with potential recall bias. Unfortunately, while biomarkers of exposure can reduce such bias, these measures rarely can reconstruct past exposure and may be affected by the *current disease status*, which may be one of the great challenges of retrospective studies. A fundamental requirement of a case–control study design is that cases and controls should be selected from the same population [20]. Population-based incident cases allow investigators to maximize the generalizability of the findings. Selection bias is generally a concern in case–control studies[21]. While the assessment of gene-environment interactions will not be subject to selection bias if participation does not differ by genotype conditional on exposure and disease status [22]. This assumption may seem reasonable for most genes and exposures, with the possible exception of (1) alleles that influence behavior, such as *aldehyde dehydrogenase* polymorphisms and alcohol exposure [23]; or (2) population stratification; or (3) alleles and exposure risk factor that influence disease detection. For example, in populations where prostate-specific antigen (PSA) screening is commonly performed, higher PSA levels often trigger for prostate biopsy and may increase early diagnosis of prostate cancer [24,25]. Differential prostate cancer screening and detection with respect to obesity [26,27] and PSA associated genes [28,29] may cause selection or detection bias. For fatal diseases, since only some of the incident cases may be available for interviewing, survivor bias can occur if genotypes or exposure status differ by survival time.

Observational epidemiological studies often suffer from confounding bias due to measured and unmeasured confounders. An example of genetic confounding bias is population stratification. Population stratification can occur in ethnically mixed populations and can lead to spurious (i.e. non-causal) associations if both the baseline disease incidence and the allele frequency vary by ethnicity [30]. Consider the hypothetical example given in Additional file 1: Table S1. In this example, there are a total of 2400 subjects in each of the two populations. Within each population, the OR associated with a genotype, e.g. assuming a dominant model, G=1 versus G=0 is 1. However, if one ignores the population labels and pools the data of the two populations together the data, the OR becomes 1.8. This spurious association between gene and disease is attributed to the fact that most cases are from population 1 and most controls are from population 2.

Although most bias due to population stratification can be eliminated by following the rules of well-designed, well-conducted study and matching or adjusting on ethnicity, this may not apply to populations whose ancestors recently mixed, such as African or Hispanic Americans [31,32]. Several genomic control approaches have been used to attempt distinguishing the ethnicity by genotyping markers that are unrelated to disease and known to have different allele frequency in ancestral populations [33,34]. Fully distinguishing the observed association from population stratification bias, can be achieved by replication of consistent findings from multiple well-designed studies in different populations or family-based study design which preclude stratification [32]. Unlike the traditional case–control studies based on unrelated individuals, family-based studies are immune to population stratification bias [35,36]. Family-based studies of gene–environment interaction sometimes may be more powerful than population-based studies [37]. However, the application could be limited by shared environment among family members and the difficulties to collect DNA samples from family members than from unrelated cases and controls, especially for long latency or late-onset diseases. Family-based studies generally have less power for genetic main effects than do case–control studies. Besides, family-based studies usually collect environmental exposure information retrospectively and may have similar problems in exposure assessment as retrospective case–control studies. The over sampling of intact families would also not be expected to represent social environments in the general population. Another approach is to use the *case-only* method to study gene-environment interaction. This approach does not allow evaluation of the main effects of the genotype alone or the exposure alone, but only their interaction [38,39]. The case-only design requires an assumption of gene-environment independence in the general population [40,41].

The *prospective cohort* study requires study subjects to be recruited before the onset of disease. This approach has the advantage of prospective collection of environmental information and biomarkers, which both precede the disease and will be unaffected by recall bias [42]. Effective follow-up should minimize selection bias secondary to attrition, one can estimate the disease incidence rate, and the inference for an underlying cohort is often well defined. Analysis of data from cohort studies is subject to bias due to loss of follow-up. As incidence rates of most diseases are low, even with many years of follow-up a cohort study often requires collection of an *extremely* large number of individuals before the onset of disease and a sufficient follow-up time, which simultaneously lead to extraordinary cost increase (i.e. by completing follow-up and data collection, including the data of baseline characteristics, exposure, and genotyping data). Hence, prospective studies are considerable challenges for diseases with low incidence rate. Risk-based sampling is being used to increase the power of prospective studies by enrolling first-degree relatives of probands, such as the Sister Study for breast cancer risk [43,44] or the on-going Early Autism Risk Longitudinal Investigation (EARLI) study for autism risk. For common pediatric diseases such as asthma, obesity, and some adverse birth outcomes, a prospective cohort study will be extremely valuable to identify environmental risk factors as well as evaluate gene-environment interaction mechanisms [45,46]. Prospective cohort studies on a national scale [47] or by pooling data from existing prospective cohorts [48] should be conducted to ensure sufficient power in gene-environmental studies. The U.S. Congress, through the Children’s Health Act of 2000, authorized the National Institute of Child Health and Human Development (NICHD) “to conduct a national longitudinal study of environmental influences (including physical, chemical, biological, and psychosocial) on children’s health and development” [49]. The National Children’s Study is a 21-year prospective cohort study of 100,000 US-born children. Environmental exposures, including chemical, physical, biological, and psychosocial exposure, will be assessed repeatedly during pregnancy and childhood in children’s homes, schools, and communities. The National Children’s Study will provide great opportunities to gene-environment interactions for common pediatric diseases.

## Measurements of exposure and effects by life stage

### Exposure biology

Measurement errors, such as misclassification of genotypes or exposure status, can exist regardless of study design. Measurement of environmental exposures have been a great challenge in epidemiologic studies due to the complex pattern of long-term exposures and the need to collect accurate and repeated individual exposure data in large populations [50]. Misclassification of exposure generally leads to attenuation of the main effects when the error is non-differential [51]. Non-differential misclassification can also bias away from the null in some circumstances, including (1) if the exposure is multilevel (>2 levels), the intermediate levels of exposure could be biased away from null [52,53]; (2) if the misclassifications are correlated with other errors [54,55]; (3) if the measured exposure do not change monotonically with the true exposure [53,56]. However, in the estimation of multiplicative gene-environment interaction effect, Garcia-Closas *et al.*[57] showed that under a set of conditions typically satisfied in studies of gene-environment interactions, both differential and non-differential misclassification of a binary environmental factor biases a multiplicative interaction effect toward the null value. These conditions are that: (1) the environmental exposure is independent of the genotype among the controls, and (2) exposure misclassification is non-differential to the genotype. This result is also true for misclassification of genetic factors.

The use of questionnaires for exposure assessment relies on personal memory and has the potential for recall bias. Several technologies have been developed to improve measurements of environmental exposures. To incorporate qualitative and quantitative changes of environmental exposures, such as atmospheric conditions and topography, over time and space, as well as individuals’ diverse demographic characteristics, lifestyles, activity patterns, geographic information systems (GIS)/global positioning system (GPS), personal monitoring, and biomonitoring are now being used in environmental epidemiology. Combined geospatial tools with statistical models allow investigators to model the transport of the pollutants from source to residence, e.g., using wind speed, temperature, and traffic density in addition to measurements from the central site, to estimate an individual-level exposure as well. Direct exposure monitoring includes personal monitoring by measuring toxics on or near the body, such as measuring air pollutants exposure levels at the breathing zone, or by sampling biological properties, such as the measurement of urinary 1-hydroxypyrene (1-OHP) as a biomarker of short-term polycyclic aromatic hydrocarbon (PAH) exposure [58]. Biomarkers of exposure are biological indicators of exogenous agents within the biological system, or other event in the biological system related to the exposure. With stringent quality control, these monitoring data hold great promise for improving exposure assessment by providing objective individual-level measurements. Biomarkers can be used to reflect the effects of earlier exposures and the association between exposure and disease at the molecular level [4-6]. Examples of intermediate biomarkers include chromosomal alterations, DNA, RNA and protein expression. In response to exposure, patterns of gene expressions, proteins, or metabolic profiles in cells and tissues change can serve as biomarkers for exposure or effect. These dynamic features however, make their interpretation in human studies challenging. Single measurement may not be reliable especially in those investigating long-term chronic effects. Incorporating long-term monitoring data with different exposure assessment techniques is needed to provide an integrated view of exposure in complex exposure–disease relationships [59,60].

### Developmental life stage and gene-environment interactions

Measuring environment has added complexity beyond issues of measurement error or selection bias. Even measuring cumulative exposure prospectively may be insufficient to capture gene-environment interaction. This is because human development occurs in life stages during which gene expression undergoes radical yet temporary changes. Environmental exposures might alter the timing of normal developmental regulation of gene expression or the gene product expressed solely at a specific life stage may interact with the environmental exposure. In particular, during prenatal life and childhood, critical biological events occur that establish the number, connections and proper function of cells within given tissues. As an example, changes in gene expression could be modulated through DNA promoter methylation or chromatin remodeling, which may be induced by environmental exposure, particularly in early development [16]. Toxicological studies show that the central nervous system is especially vulnerable to toxic injury [61] and epidemiological studies clearly show an association between adverse neurodevelopment and *in utero* exposure to chemicals such as methyl mercury [62,63], PCBs [64], while exposure later in life demonstrates less toxicity. Epidemiological studies of chemicals typically show a large variance around the effect estimate for the dose–response relationship. While many factors contribute to this variance, including measurement error in exposure and/or phenotype, it is likely that the timing of the exposure and variant genetic factors that modify the response to toxicants contribute significantly to the observed variance. Genetic variants that produce gene-environment interactions may only do so when the exposure corresponds to a critical developmental window during which that gene is highly expressed. This is a fundamental concept in developmental biology that is often overlooked in epidemiologic studies. Indeed the concept of fetal origins of adult diseases demonstrates the critical nature of exposure *timing* in producing later health effects (e.g., the association of maternal smoking during pregnancy and reduced fetal growth [65], obesity [55], decreased lung function [66] and diabetes [67] in the offspring). Although a prospective study can address timing of exposure in a clearly unbiased manner, it is still challenging to assess the details of exposure timing and risk as the critical window likely differs for different phenotypes and for different exposures. It is also not possible to know with certainty what the critical exposure window is *a priori* (i.e. *in utero* vs. childhood vs. puberty). The difficulties in assessing the effects of exposure by timing present in carefully designed observational studies and even trial results. An example is the initial report from Women’s Health Initiative (WHI) randomized trial and epidemiologic data on the risk of coronary heart disease (CHD) and the menopausal hormone therapy. Large observational studies include Nurses’ Health Study (NHS) suggested a reduced risk of CHD among postmenopausal hormone therapy [8,68] while WHI randomized trial found increased risk of CHD among women assigned to the menopausal hormone therapy compared to the placebo group [69]. Hernán et al. re-analysis of the Nurses’ Health Study and concluded that most of the difference could be attributed to the age distribution at the time of initiation of hormone therapy and length of follow-up [70].

Unfortunately, for most adult diseases, an unbiased reconstruction of childhood exposure is difficult, if not impossible. Thus, a major limitation of adult epidemiologic research will continue to be the inability to reconstruct childhood factors that predict disease. At least some of the difficulty in finding gene-environment interactions for adult disease is likely that the relevant exposure may have occurred in childhood, and a measure of cumulative exposure, while preferable to cross-sectional measures, cannot capture exposure during the critical developmental life stage predisposing to disease.

## Statistical analysis issues for gene-environment studies

### Longitudinal studies

In order to incorporate exposure effects by life stage, gene-environment interaction may be conceived as a 3-way interaction, in which the time of the exposure is the 3^rd^ factor. In general the gene-environment interaction as a function of time can be modeled by considering a general nonparametric model

$$Y_{\mathrm{ij}}=f\left(G_{\mathrm{ij}},E_{\mathrm{ij}},t_{\mathrm{ij}}\right)+e_{\mathrm{ij}},$$

where Y_ij_ is the response of interest of the i-th subject at the j-th time point t_ij_; G_ij_ and E_ij_ are the genetic and environmental covariates measured at t_ij_, and e_ij_ are random errors. Here the function f(.) models the combined effect of gene, environment and any possible interactions as function of time. Note that the formulation above can incorporate multiple genetic and environmental variables and thus has potential to model gene-gene interactions as well as gene-environment interactions involving several genes as well. For such general model of longitudinal data, Zhang [71,72] presented multivariate adaptive spline smoothing based estimation methods. For high-dimensional data, such as GWAS studies directly applying such methods for a large number of SNPs is undesirable. Zhu et al. [73] adapted the multivariate spline methodology for GWAS: Specifically, the procedure starts by starting with a model containing only intercept (the simplest model) and then gradually growing the model by adding terms (e.g., individual SNPs, SNP-SNP interaction) that minimizes a weighted least squares criteria. Finally the end model is selected via a backward step by deleting one least significant term at a time from the model.

Another popular and useful approach for modeling factors that change over time is the varying coefficient modeling strategy. Specifically for G-E interaction, one can consider the time-varying coefficient model

$$\begin{array}{cc} \;Y_{i}\left(t_{\mathrm{ij}}\right) & =\beta_{0}\left(t_{\mathrm{ij}}\right)\;+\;G_{\mathrm{ij}}\beta_{G}\left(t_{\mathrm{ij}}\right)\;+\;E_{\mathrm{ij}}\beta_{E}\left(t_{\mathrm{ij}}\right)\\ & \;+\;G_{\mathrm{ij}}{*E}_{\mathrm{ij}}\beta_{\mathrm{GE}}\left(t_{\mathrm{ij}}\right)\;+\;e_{\mathrm{ij}}, \end{array}$$

where t_ij_ denotes the time point for the j-th measurement of the i-th subject; G_ij_ and E_ij_ are the genetic and environmental covariates measured at t_ij_; β_G_(.), β_E_(.) and β_GE_(.) are unknown gene, environment and G-E interaction effect, respectively, depending on time. Note that this is a generalization of the conventional two-way G-E interaction model Y_i_(t_ij_) = β_0_ + G_ij_β_G_ + E_ij_β_E_ + G_ij_E_ij_β_GE_ + e_ij_ with non-time-varying effects. Depending on the data at hand, one could also consider different version of this model in various ways, e.g., β_G_(t_ij_) = β_G_ corresponds to the model where one assumes that only the intercept, the environment effect and G-E interaction effect vary over time but the gene effect does not. There is a rich literature on varying coefficient models discussing estimation and testing procedures, e.g., Hoover, Rice, Wu and Yang [74] and Wu and Chiang [75] among many others. The coefficient β_GE_(.) reflects the G-E interaction effect as it changes over time. Thus, if the G-E interaction is prominent at a specific window of time but dormant in others, plotting this coefficient function over time could potentially reveal such patterns.

### Case–control studies

Case–control studies are commonly used in studying for genes and environment. Case–control studies sample disease subjects (cases, D=1) and healthy subjects (controls, D=0), and retrospectively collect information about genes (G) and environment (E). The description of a simple case–control study is given in Additional file 1: Table S2, where both E and G are binary. The data from a case–control study can be used to compute three odds ratios (ORs), using subjects who are unexposed and have typical genotypes as they occur in nature (also known as wild type) (E=G=0) as the reference group: OR_11_ for subjects with both the gene and the exposure (E=G=1), OR_10_ for subjects with only the exposure (E=1, G=0), and OR_01_ for subject with the only gene (E=0, G=1). Then under the multiplicative interaction model, the null hypothesis of no interaction can be written as OR_11_ = OR_01_ × OR_10_. Thus, to test for GxE interaction, one defines the interaction odds-ratio as OR_**I**_ = OR_01_ × OR_10_/ OR_11_ and tests for H0: OR_**I**_ = 1. From Additional file 1: Table S2, the sample log(OR_I_) can be estimated as log(bche/adfg), and one can then construct a Z-statistic to test for H_0_ (see for example, [76]).

Logistic regression is commonly used for analysis of case–control studies, especially in the presence of covariates. A typical logistic model for assessing gene-environment interaction is

(1) $$\begin{array}{cc} \;\mathrm{logit}\left(p\right)= & \beta_{0}\;+\;\beta_{1}G\;+\;\beta_{2}E\;+\;\beta_{3}G\;*\;E\;\\ & +\;\beta_{4}X \end{array}$$

where p is the population disease probability and X is a vector of covariates. As subjects are sampled based on the case–control status and cases are over-sampled, the likelihood depends on distribution of the independent variable (G, E and X) in the population and the case–control sampling probability. Hence the intercept β_0_ cannot be estimated from the case–control sample. However, Cornfield [77] and later Prentice and Pyke [78] showed that one can estimate all the regression coefficients β except for the intercept using the ordinary logistic regression likelihood as if the data were obtained in a prospective study.

Under model (1), the OR of (G, E) versus (G_0_, E_0_) is then given by exp{ β_1_(G - G_0_) + β_2_(E - E_0_) + β_3_(GE- G_0_E_0_)}. In the presence of gene-environment interaction, the OR of disease and gene depends on exposure. For example, consider the case when both G and E are binary. The covariate X adjusted OR of D and G in the unexposed group (E=0) is exp(β_1_) and the OR of D and G in the exposed group (E=1) is exp(β_1_+ β_3_). The interaction OR_I_ = exp(β_3_). The null hypothesis H_0_: β_3_ =0 constitutes a no gene-environment interaction. Note that no assumption about the distribution of gene (G), environment (E) and covariates X, e.g., independence of gene and environment, is made in logistic regression.

Several advanced models have been developed to incorporate gene-environment interactions. Selinger-Leneman et al. [79] explored the conditions under which accounting for gene-environment interaction enhances the ability to detect the genetic effects in complex diseases. Chatterjee, et al. [80] developed a maximum score based testing procedure for main gene effects in the presence of possible gene and environment interaction using parametric models. Kraft et al. [81] applied a two degree-of-freedom likelihood ratio test for the association between a disease and a genetic locus, allowing for the possibility that the genetic effect may be modified by an environmental factor. Maity et al. [82] developed more flexible statistical tests for genetic main effects in presence of possible gene-gene and gene-environment interactions using a semiparametric method.

Nevertheless, one should be aware that the case–control method may not be applicable for association studies in some situations, such as in the presence of population stratification that can not be estimated from the data. It is useful to complement case–control studies with family studies using genetic analytic techniques such as segregation and linkage methods [83].

### Case only studies

An important matter in case–control studies is the choice of control group. An inappropriate choice of controls, e.g., hospital based controls or shared controls for different studies, may result in erroneous findings, e.g., due to population stratification. To address this problem, several approaches have been developed, see e.g., [40]. One of these approaches to assess G-E interaction is the case-only design where one uses only cases (D=1).

A key assumption to study G-E interaction on D in the case-only design is that the distributions of gene and environment are independent. Examples of such situations are the cases when an environmental factor is not directly controlled by individual behaviors, e.g., air pollution. Specifically, in the absence of covariates, under model (1), assuming rare disease, Pr(D=0 | G, E) is approximately 1. Assuming that G and E are binary and independent in the population, it can be shown that the OR relating exposure and genotype in cases only is

$$\begin{array}{c} \mathrm{pr}\left(G=1,E=1|D=1\right)\;\mathrm{pr}\left(G=0,E=0|D=1\right)/\\ {}[\mathrm{pr}\left(G=0,E=1|D=1\right)\;\mathrm{pr}\left(G=1,E=0|D=1)]={\exp(\beta }_{3}\right). \end{array}$$

This corresponds to the OR in a simple 2x2 contingency table (Additional file 1: Table S3)

Thus, one can estimate the effect of the G-E interaction term approximately correctly without performing a logistic regression of D. This approach can also be applied in logistic models in the presence of covariates [39]. Under the assumption of the independence of gene and environment, the case-only analysis yields a smaller standard error when estimating the interaction term β_3_, thus increasing power to detect GxE interaction [39]. Umbach and Weinberg [84] conjectured that imposing the gene and environment independence assumption in studies where controls are available could also improve precision for estimating main effects. They also investigate the power gain in detecting GxE interaction via simulation studies and find that in several parameter configurations considerable precision advantages can accrue by estimating the interaction term using G-E independence assumption. They find that sometimes the variance of the interaction term can be reduced by more than two-fold, even near the null value β_3_=0. Thus, in situations where the key independence assumption is met, a study analyzed with G-E independence assumption may need considerably fewer subjects than one analyzed with the full model without G-E independence assumption to achieve the same power for detecting gene-environment interaction. Several researchers exploit the assumption of gene-environmental independence in the population to develop more powerful statistical tests for gene and environment interactions in more complex settings, see e.g., [84-86].

However, one should exercise caution when applying case-only analysis, as it makes a strong assumption that G and E are independent in the population, possibly conditioning on covariates. If the distribution of G and E depend on each other, the case-only design will yield a biased estimate of the interaction term β_3._ In addition, it only estimates the interaction term β_3_ and cannot estimate the main effects β_1_ and β_2_. In practice, the assumption of G-E independence in the population may not hold. For example, the genetic variants in a smoking pathway may affect the degree of addiction. In such scenario, a case-only study for studying the effects of genes and lung cancer risk would not be applicable. Further, the validity of a case-only study also hinges on the assumption that there is no hidden population stratification in the study population. Wang and Lee [87] showed that if a population stratification exists, then case-only studies may be biased, and the bias involves the coefficient of variation of the exposure prevalence odds, the coefficient of variation of the genotype frequency odds, and the correlation coefficient between the exposure prevalence odds and the genotype frequency odds. In other words, a case-only study may be biased if a systematic difference is present in either genotype frequencies or exposure prevalence between subpopulations.

### Case-parent and case-sib design

In a ‘case-sib’ design, each case is matched to one or more unaffected siblings [88-90]. Compared to the case–control design, this design has the advantage that cases and controls are perfectly matched on the ethnic background, thus this design reduces the bias due to population stratification.

In the ‘case-parent’ design, the parents of cases are used as a sort of control group to study genetic markers that could be associated with disease risk or be in linkage disequilibrium with alleles at a neighborhood locus. Genotypes are obtained from each case and his/her two parents, while environmental data are required only from cases [41]. Similar to the case-sib design, this design provides a perfect control for ethnic confounding. The main effect of environmental factors cannot be assessed in the case-parent design, but analysis of genetic main effects and G*-* E interactions can be conducted. Umbach and Weinberg [91] proposed an association test, which examines the joint effects of gene and environment using case-parent trios. The case-parental control method requires the availability of genotypic information on both parents of cases, although the EM algorithm can be used to maximize the likelihood if some genotypes are missing and the method has been extended to situations where only one parent is available [92]. Witte et al. [90] and Gauderman et al. [89] compared the relative efficiency of the case-sib and case-parent designs to the matched case–control design for estimation of genetic main effects. They also provided some comparisons of efficiency for estimation of the G × E interaction effect. They found that because of overmatching on genotype, the use of sibling controls leads to estimates of genetic relative risk that are approximately half as efficient as those obtained with the use of population controls, while relative efficiency for cousin controls is approximately 90%. However, they also find that for a rare gene, the sibling-control design can lead to improved efficiency for estimating a *G* × *E* interaction effect.

### Genome-wide association studies

A genome-wide association study involves scanning tens of thousands of genetic markers (SNPs) across the genome to identify the genetic variations that are associated with a disease or a trait [93,94]. Such studies are particularly useful in finding common genetic variations that contribute to common and complex diseases, such as heart disease, cancer, and diabetes. Compared to linkage analysis, GWAS can be more powerful in detecting genes associated with modest increases in disease risk [95]. In the past few years, GWAS have been successful in identifying over a hundred common genetic variants that are associated with complex diseases (http://www.genome.gov/gwastudies).

In a traditional case–control GWAS, one observes a disease outcome D, environmental exposure E, and the genotypes of M SNPs spanning the genome, with g_1_, g_2_, …, g_M_ denoting the genotypes at the M loci. Illumina and Affymetrix provide common genotyping platforms for GWAS, where the genotypes of a million or more SNPs can now be simultaneously measured. Several models can be used for the pattern of inheritance of the genetic susceptibility. Under the dominant model, subjects with genotype g = AA or Aa are genetically susceptible, that is, they are at either increased or decreased risk compared to the baseline group (g = aa). This structure can be captured by defining the genetic covariate G such that G = 0 for g = aa, and G = 1 for g = AA or g = Aa. Under the recessive model, we have G = 1 for g = AA and G = 0 otherwise. Under the co-dominant model, one can use two dummy variables or an additive model (G=0,1,2) to model the genetic effect. Let p and q = 1- p be the probabilities of observing A and a respectively. Assuming Hardy-Weinberg equilibrium, the distribution of genotypes g in the population is given by pr(g = AA) = p^2^, pr (g = Aa) = 2pq, and pr(g = aa) = q^2^. Hardy-Weinberg equilibrium should be checked when the genotype data are cleaned.

GWAS studies have primarily focused on detecting the main gene effect by fitting the traditional logistic main effect model for each SNP G_j_ separately as

(2) $$\mathrm{logit}\left(p\right)=\beta_{0}+\beta_{1}G_{j}+\beta_{2}E+\beta_{4}X,$$

where X is a vector of covariates and often also includes a few principal components to control for population stratification [96]. A correction for multiple comparisons, such as the Bonferroni correction, or modified Bonferroni correction [97], is often used to control for the genome-wide type I error. Several multi-locus tests have been proposed to improve the power in GWAS studies[98]. Top SNPs from GWAS are then selected for validation in independent samples.

To study gene-environment interaction in GWAS, one can fit model (1) for each SNP separately and test for H_0_: β_3_ = 0 and use the Bonferroni correction to adjust for multiple comparisons. A main challenge in using GWAS to test for G-E interaction is that most GWAS have limited power to detect gene-environment interaction on the genome wide scale after accounting for multiple comparisons. One might consider using the case-only analysis to increase the analysis power. However, the case-only analysis relies on the strong assumption of the independence of gene and environment in the population, which might be not reasonable across all SNPs that are scanned in a GWAS.

Several approaches have been recently proposed to improve power for detecting gene-environment interactions in GWAS. Kraft et al. [81] proposed to screen for top genes in the presence of possible gene-environment interactions using a 2-df test for testing for the main genetic effect and G×E interactions jointly. They showed that under a variety of parameter settings, the 2-df test was often more powerful than a test of the main effect or the traditional test for G×E interactions.

Assuming binary E, Murcray et al. [99] proposed a two-step approach where they first use a likelihood ratio test of the association between G and E based on the logistic model pr(E = 1 | G) = a_0_ + a_1_G and test for H_0_: a_1_=0. This corresponds to the standard case-only test for the G×E interaction. One then screens for the significant genes with p–values below a threshold. In the second step, the screened SNPs are then tested using the standard G x E interaction test under model [1] with correction for multiple comparisons. They showed this two-stage test is more powerful than the standard one-stage test for the gene-environment interaction (H_0_: β_3_=0) using model [1]. The added power of this two-stage procedure derives from the fact that the multiple comparison in the second step is performed only based on the genes chosen in the first step, not the entire set of genes. As shown by Murcray et al., this two-step method can be almost twice powerful than the traditional one-step procedure if the G-E independence assumption is valid for a large fraction of G-E combinations under study. However, the power gain of the procedure diminishes as the total number of genetic markers increases [100].

Mukherjee and Chatterjee [101] proposed a 1-stage inferential procedure on G-E interactions using an empirical Bayes-type shrinkage estimation approach. They estimate the interaction using a weighted average of the case-only and case–control estimators where the weights are based on the difference between the two estimators and the variance of the robust case–control estimate. This estimator is shown to be robust to the departure from the G-E independence assumption. The associated test can gain efficiency and power when the assumption of G-E independence in satisfied in the underlying population but also preserves Type I error when the independence assumption is violated.

### Cohort studies

In prospective cohort studies, a sample of healthy subjects in a pre-specified cohort of subjects are recruited, environmental and lifestyle data and Biological samples, such as blood, are obtained at baseline (the start of the study), and the subjects are then followed prospectively over time for disease onset or quantitative traits*.* Questionnaire data and biological sample may be also updated over time. As Clayton and McKeigue [102] state, “The rationale for setting up cohort studies of genetic effects on disease risk is based on the argument that, because cohort studies can measure environmental exposures before disease onset, they are better than the case–control design for study of gene-environment interactions. Study of such interactions is thought to make detection of genes that influence disease risk easier, to allow individuals at high risk to be identified for targeted intervention, and to advance understanding of biological pathways leading to disease.”

For binary D, E, and G, the data layout for a cohort study is similar to that of a case–control study in Additional file 1: Table S2, except that, unlike case–control studies, one can now estimate disease risks and subsequently estimate relative risks (RRs). Parallel to the odds ratio calculations in case–control studies, one can define four RRs. For example, using the non-exposed and non-high-risk genotype (G=E=0) as the reference group: RR_11_ is the RR comparing the exposed and high-risk genotype group (G=E=1) and is estimated as h(a+c)/(f+h)a, similarly one can define RR_10_, RR_01_, and RR_00._ Under the multiplicative interaction model, the null hypothesis of no G-E interaction can be written as H_0_: RR_11_ = RR_01_ × RR_10_. This is equivalent to testing H_0_: OR_11_ = OR_01_ × OR_10_ and can proceed with logistic regression.

One major limitation of cohort studies is that rare events will not occur at sufficient frequency so that most cohort studies may not record sufficient numbers of cases for rare diseases and might have only marginal power for common diseases [102]. Cohort studies can be used to study gene effects and gene-environment interactions for disease progression and censored time-to-event data using survival analysis techniques, e.g., the Cox model [103], and for longitudinal phenotypes using mixed models and GEEs [104].

### Nested case–control design and case-cohort studies

Epidemiologic cohort studies and disease prevention trials typically require the follow-up of several thousand subjects for a number of years before yielding useful results, and hence can be prohibitively expensive. To address this issue, a pseudo case–control design can be used to reduce the number of subjects for whom covariate data are required (see for example, [105-108]), where each subject developing disease is matched to one or more subjects without disease at the same point in 'time' using incidence-density sample. Henceforth, relative risks are estimated using a matched case–control analysis. In this setup, one only requires the covariate measurements for only cases and their matched controls. This is the so-called 'case-control nested within a cohort' design.

However, intuitively the alignment of each selected control subject to its matched case could be inefficient, since that subject may also properly serve as a member of the comparison group for cases occurring at a range of other times. In the context of a disease prevention trial, it is often desirable to have a subset of the trial cohort for whom covariate data are analyzed on an ongoing basis in order to monitor intervention effectiveness and compliance. The case–control approach is not well suited to this purpose since covariate histories are only assembled following case occurrence. As an alternative, Prentice [78] proposed a ‘case-cohort’ design which involves the selection of a random sample (or a stratified random sample) of the entire cohort, and the assembly of covariate histories only for this random subcohort and for all cases. The subcohort in a given stratum constitutes the comparison set of cases occurring at a range of failure times. The subcohort also provides a basis for covariate monitoring during the course of cohort follow-up. Very similar designs have also been proposed by Kupper, McMichael & Spirtas [109] and Miettinen [110]. These more efficient designs have started being used to study gene-environment interactions in cohort studies [111].

The statistical efficiency for a case-cohort study over a nested case–control study is small. Wacholder [112] pointed out that nested case–control designs have a small to moderate advantage for studies with substantial late entry or censoring. Case-cohort studies gain small advantage in studies with little late entry or censoring. However, a major practical advantage of the case-cohort studies is the ability to use the same subcohort for several outcomes such as different subtypes of disease [112]. If one intends to compare the risk factors of different outcomes then adjustments of significance levels and confidence intervals are required due to multiple comparisons and to account for possible correlations between outcomes [113]. However, if the main focus is on the evaluation of risk factors for each disease separately then no such adjustment is required.

### Two-stage designs and biased sampling

In many situations, the exposure of interest and the disease endpoint can both be rare and studies of their relationship between them require a very large number of samples, and hence can be very expensive. In such cases, a two-stage design, originally proposed by White [114], can be employed. A major assumption in this scenario that the exposure information is already available for a large sample of controls and cases in the screening stage. Complete covariate and genotype information is then collected only on a subsample, where the sampling fraction can depend jointly on disease and exposure status. For example, in case of a rare exposure, one can oversample those who are more likely to have exposure and perform the genotyping on a more informative subset of subjects. A similar approach can be taken where a specific rare genotype is of interest and exposure is expensive to record. White [114], assuming the exposure and disease status are both binary, presented a procedure to derive valid estimates of odds ratio by incorporating the information from the first both stages sample and the sampling proportions for the second stage. Cain and Breslow [115] extended this approach by allowing for a multilevel exposure variable, and any number and type of any covariates. There is a large recent literature on analysis of two-stage case–control designs using more efficient inverse probability weighted estimation procedures and semiparametric efficiency procedures [116]. Weinberg and Wacholder [117] developed designs of case–control studies with biased sampling for more general cases. They developed and presented analytic techniques and estimation procedures. They show that unbiased estimation procedure of the main and the interaction effects are possible assuming given the sampling fractions are known for the second stage sampling. From the simulation studies of Weinberg and Wacholder, it is seen that the effect of the screening/matching factor in the stage 1 sampling can often be estimated with better precision compared to completely sampling. In addition, the main and interaction effects can be also estimated more efficiently compared to random sampling. The advantage of this design appears to improve the efficiency of estimation of the interaction coefficient; the efficiency gain could be as large as 250%. The efficiency gain is however dependent of the odds ratio relating the exposure and genotype to the disease.

### Power and sample size considerations

Gene-environmental studies often require large sample sizes to detect interactions compared to studies for detecting main gene and environmental associations. Thus, power and sample size considerations are critical. There have been several publications about sample size and power calculations in G-E studies (Table 1). The software QUANTO developed by Gauderman [37] is convenient for power and sample size calculations for a range of gene-environmental designs.

**Table 1:** Summarized publications regarding sample size and power calculations in gene-environment studies

**Source**	**Design**
Yang et. al. [118]	Case-only
Cai and Zheng [119]	Case-cohort
Schaid [41]	Matched case–control
Gauderman [37]	Case-sibling
	Case-parent
Lubin and Gail [120]	Unmatched case–control, Multivariate regression models for odds ratio
Hwang et al. [121]	Unmatched case–control, binary genetic and environmental factors
Foppa and Spiegelman [122]	Unmatched case–control, binary genetic factor and an environmental exposure with multiple categories

## Discussion

With advancement in human genetics and risk assessment, current research has shown that the interplay between genes and environment is critical to disease risk and progression. Consequently, more research efforts need to be directed towards investigating the genetic basis of individual susceptibility and the role of the genome and epigenome, to various environmental agents. The methodological issues raised above are focused on the “how to” approaches to assessing gene-environment interactions.

All individuals are exposed to a variety of hazardous agents and chemicals in the environment. However, genetic pathways are thought to have evolved for minimizing the adverse effects from these environmental insults. Genes expressed in these pathways, referred to broadly as environmentally responsive genes, exhibit heritable variability that may be associated with altered efficiency of the pathway. Hence, gene-environment investigation needs to go beyond individual genes to investigate the roles of genetic pathways and networks.

Several research programs were launched to promote and facilitate research in environmentally responsive genes. In the 1990’s, the National Institute of Environmental Health Sciences (NIEHS), of the U.S. National Institutes of Health, initiated a multiyear project entitled the NIEHS Environmental Genome Project (EGP). The focus of the NIEHS EGP is on common sequence variations, referred to as genetic polymorphisms, in environmentally responsive genes. The NIH-wide Genes, Environment and Health Initiative (GEI) was launched in February 2006 to support research that will lead to the understanding of genetic contributions and gene-environment interactions in common disease. Numerous scientific advances have been made through these initiatives.

More advanced statistical and computationally efficient methods need to be developed to investigate the interplay of genes and environment in human diseases. Data integration is becoming more and more important. More interdisciplinary research by integrating molecular biological knowledge, environmental sciences, bioinformatics and computational biology, and statistical and computational methods is likely to advance research in genes and environment. More research is needed in several emerging research areas in genes and environment, such as exposure biology for identifying new biomarkers for better measuring exposures, mediation (causal inference) analysis, e.g., for effects of environment of disease phenotypes through epigenetic markers, statistical methods for high-dimensional data analysis for genes and environment, and risk prediction using genes and environment. It should be noted that the process of translating genetic and ‘omic research into practice in environmental and occupational health is considered to be in an early phase. Thus, most research findings from genetic susceptibility studies should be communicated with caution to the general public at this time. Policy research on genes and environment deserves more attention.

## Conclusion

In conclusion, we are entering an exciting period of research and knowledge generation about gene-environment interactions. The potential for combining basic bench work with human population studies opens up many opportunities to examine the health effects of complex environmental exposures. The challenges for the next decade for human population work in this field include maintaining rigorous epidemiologic study design, improving environmental exposure analysis, advancing genomic technology and knowledge, and expanding the necessary analytic and computational tools for high-throughout “-omic” and environmental data, and the concomitant policy and ethical implications.

## Misc

Chen-yu Liu and Arnab Maity contributed equally.

## Competing interest

None of the authors has any actual or potential conflicts of interest.

## Supplementary Material

Additional file 1 **Table S1.** Example of population stratification. **Table S2.** OR calculations for G-E case-control studies. **Table S3.** OR calculations for case-only (D=1) studies. Click here for file
