# Supplementary material for: Design and analysis issues in gene and environment studies
Source: Environ Health. 2012 Dec 19;11:93. doi: 10.1186/1476-069X-11-93 (PMC3551668; doi:10.1186/1476-069X-11-93)
Supplement: Additional file 1 — Table S1. Example of population stratification. Table S2. OR calculations for G-E case-control studies. Table S3. OR calculations for case-only (D=1) studies. [file 1476-069X-11-93-S1.doc]

Supplemental Material, Table 1: Example of population stratification

|  | Population 1 | | Population 2 | | Total |
| --- | --- | --- | --- | --- | --- |
|  | G=1 | G=0 | G=1 | G=0 |  |
| Case | 2 | 14 | 4 | 4 | 24 |
| Control | 1 | 7 | 8 | 8 | 24 |
| Total | 3 | 21 | 12 | 12 |  |

Supplemental Material, Table 2: OR calculations for G-E case-control studies

| Case-control study | | | | |
| --- | --- | --- | --- | --- |
|  | E = 0 | | E = 1 | |
| Disease status | G = 0 | G = 1 | G = 0 | G = 1 |
| D = 0 (controls) | a | b | e | f |
| D = 1 (cases) | c | d | g | h |
|  | OR_00_= 1 | OR_10_=ad/bc | OR_01_=ag/ce | OR_11_=ah/cf |

Supplemental Material, Table 3: OR calculations for case-only (D=1) studies

| Exposure | Genotype | |
| --- | --- | --- |
|  | G = 0 | G = 1 |
| E = 0 | a | b |
| E = 1 | c | d |

OR = ad/bc
